# Optimizing Outcomes for Patients With Soft-Tissue Sarcoma Through the Multidisciplinary Medical Oncology/Radiation/Surgical Team Approach

**Published:** 2018-04-01

**Authors:** Arash Naghavi, Dave Johnson, Leah Clark

**Affiliations:** Moffitt Cancer Center, Tampa, Florida

## Abstract

A multidisciplinary team can help improve the outcomes of patients with any cancer, but particularly so for patients with soft-tissue sarcomas, as discussed at JADPRO Live. During this presentation, MD, PA, and NP colleagues from Moffitt Cancer Center reviewed the role of systemic therapy and emergence of targeted therapy for this subset of patients.

Soft-tissue sarcomas (STS) comprise a heterogeneous group of tumors arising from transformation of mesenchymal-origin cells. Collectively, they have an annual incidence of about 12,000, and about 5,000 people die of STS each year. Historically, treatment has consisted primarily of surgery and radiation, but increasingly, systemic therapy has begun to play a role in treatment, particularly with the emergence of targeted therapies. Management by a multidisciplinary clinical team offers the potential to improve outcomes, as discussed at JADPRO Live 2017 by Arash Naghavi, MD, Dave Johnson, PA-C, and Leah Clark, ARNP, of the Sarcoma Program at Moffitt Cancer Center in Tampa.

## WORKUP

"[Soft-tissue masses] come in different sizes and shapes," said Mr. Johnson. "They can be in your extremities, they can be in your trunk, they can be in your neck, they can be in your retroperitoneum. They can be pretty much anywhere in your body, so when a patient presents with a soft-tissue mass, we need to be working it up appropriately."

"We need to have an awareness that not everything is benign. Even though masses of mesenchymal-cell origin are 100 times more likely to be benign, we can have malignant cells as well. If you look at the delay in diagnosis in soft-tissue sarcomas, it’s anywhere from 3 to 6 months. We all know that the quicker we can treat a cancer, the better outcomes we can have," he said.

The basic workup of a patient with STS consists of a history and physical, biopsy, and imaging, said Mr. Johnson. The history and physical should include the patient’s age, disease status (newly diagnosed or recurrent), limb function, performance status, and wound-related issues. Patient age is a key determinant of the level of suspicion about an undiagnosed soft-tissue mass, as the likelihood of malignancy increases with older age, except in the case of rhabdomyosarcoma, which is increasingly seen in childhood. Key information from the biopsy includes tumor histology and grade, which can inform clinical decision-making. Imaging—including plain-film x-rays, magnetic resonance imaging (MRI), and occasionally computed tomography (CT)—provides essential information for staging, revealing whether the lesion is localized, as well as its depth and size.

"MRI is really the gold standard," said Mr. Johnson. "On MRI scans, soft-tissue sarcomas usually are about 4 centimeters or greater. They are dark on T1, bright on STIR signal, and bright on contrast—heterogeneic. They have a lot of different densities. There is sometimes necrosis with it, along with surrounding edema. They have a pseudocapsule, and you can trace your finger around it and say ’that’s exactly where the mass is.’ "

## PROGNOSIS

The prognosis for STS depends on patient age and comorbidities, tumor size and subtype, histologic grade, and stage. Poor prognosis is associated with age greater than 60 years, high grade, size greater than 5 cm, and positive margins after tumor resection.

**Figure 1:**
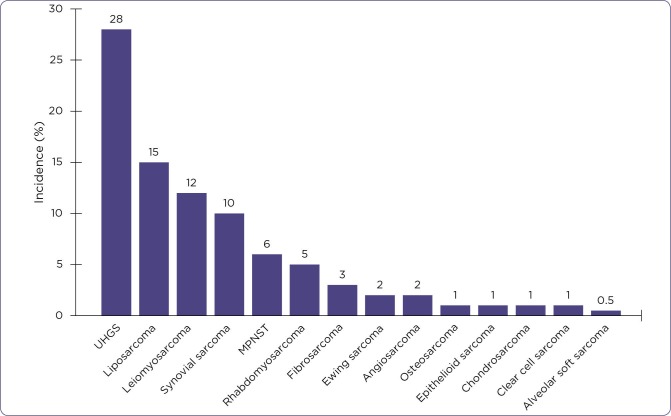
Subtypes of soft-tissue sarcoma. UHGS = undifferentiated high-grade sarcoma; MPNST = malignant peripheral nerve sheath tumor.

Further reflecting the heterogeneity of the disease, the most prevalent subtype of STS is undifferentiated high-grade sarcoma, which accounts for 27% of the annual incidence, followed by liposarcoma at 15%, and leiomyosarcoma at 12%. Most of the remaining STS disease burden is spread among nine other subtypes, none of which accounts for as much as 10% of the total (Figure 1).

Tumor size has a major impact on prognosis. Patients with tumors less than 5 cm have a 5-year survival of 75%, declining to 60% for tumors that are 5 to 10 cm, and 45% for tumors greater than 10 cm. Similarly, increasing grade has an inverse association with survival. Grade 1 (low) tumors are associated with a 5-year survival of 97%, which declines to 67% for grade 2 tumors, and to 38% for grade 3 (high) STS, the most aggressive tumors.

Not surprisingly, increasing stage is associated with worse prognosis. Patients with stage 1 STS have a 5-year overall survival of 92%, declining to 76% for stage 2, and 42% for stage 3. The vast majority of patients with stage 4 STS die of the disease, as only 3% remain alive after 5 years.

Soft-tissue sarcoma metastasizes most often to the lungs. A chest CT scan is a primary staging tool, expanding to include imaging of the abdomen and pelvis for myxoid liposarcomas, synovial sarcomas, rhabdomyosarcomas, and angiosarcomas. The lymph node is another common site of STS spread, particularly in association with rhabdomyosarcoma, alveolar sarcoma, angiosarcoma, clear cell sarcoma, epithelioid tumors, and synovial sarcoma.

## TREATMENT

Management of STS epitomizes multimodal therapy. Surgery, radiation therapy, and chemotherapy all have a role in effective therapy. Smaller, low-grade tumors often can be managed with surgery alone. For high-grade STS of the extremities, emphasis is on limb-sparing surgery—complete removal of the tumor to the greatest extent possible while preserving the greatest functionality possible.

Surgical margin status influences outcome. The minimal definition of a clear margin is one normal cell between you and the tumor. A positive margin (residual tumor) is associated with a high likelihood of recurrence. Certain types of procedures, such as removal of an entire compartment (radical resection), requires more radical surgery that necessitates removal of more normal tissue. As the extent of surgery increases, so does the risk of functional loss for the patient, said Mr. Johnson.

Data from the University of Florida showed a 4% frequency of local recurrence with radical tumor resection, increasing to 25% with wide excision, and 50% to 60% with marginal excision (Enneking, Spanier, & Malawer, 1981). Additional experience with resection that ranged between wide and marginal, plus use of adjuvant radiation therapy, resulted in a recurrence rate of about 7%, comparable to radical excision and amputation, and 25% to 30% without radiation therapy, said Mr. Johnson.

## THE ROLE OF RADIATION THERAPY

Radiation therapy for STS most often involves external beam radiation therapy (EBRT), but sometimes patients receive brachytherapy, said Dr. Naghavi. External beam radiation therapy can be administered before or after surgery, and brachytherapy is usually administered perioperatively. Reconstruction of the resulting wound may occur immediately or in a staged fashion.

As the goals of surgery incorporated limb and function preservation, interest in the potential additive benefits of radiation therapy increased. Studies comparing surgery alone vs. surgery plus adjuvant radiation therapy demonstrated an absolute improvement in local control of about 20% with either EBRT (Yang et al., 1998) or brachytherapy (Harrison, Franzese, Gaynor, & Brennan, 1993).

Preoperative EBRT offers several potential advantages over postoperative, or adjuvant, EBRT. Radiation therapy’s principal mechanism of action involves interaction with oxygen molecules to generate free radicals that cause DNA damage, said Dr. Naghavi. An unresected tumor is fully oxygenated, providing a rich substrate for the effects of ionizing radiation. Advantages of preoperative (or neoadjuvant) EBRT over postoperative EBRT include:

- Low radiation dose requirement, potentially reducing long-term toxicity (Zagars et al., 2003)
- Fewer treatment fractions, or sessions, potentially reducing cost and increasing patient convenience
- Smaller treatment volume from eliminating need to treat surgically manipulated areas
- Tumor shrinkage that may improve the chances of complete surgical resection (Robinson et al., 1992)
- Potentially improved disease control (Al-Absi et al., 2010; O’Sullivan et al., 2002; Sampath et al., 2011)

The principal downside of preoperative radiotherapy for STS is an increased risk of acute major wound complications (Sampath et al., 2011).

"[Additionally,] although it’s rare, there is the possibility that when you radiate up-front, the tumor could progress through radiation and could potentially make the patient unresectable," said Dr. Naghavi.

Use of brachytherapy follows local wide excision of the tumor. The radiation oncologist positions catheters at approximately 1 cm intervals across the plane of the tumor bed. The catheters act as the conduit for delivering the radioactive seed, which either remain in the patient for a duration of minutes to days, depending on the brachytherapy delivery technique (e.g., high dose rate [HDR] or low dose rate [LDR] technique). Catheter placement is determined after discussion with the surgeon, reaching agreement on the areas of the tumor bed that has the highest risk of residual microscopic disease. Frequently, surgeons will place clips to delineate the tumor bed when planning radiation delivery (Naghavi et al., 2017).

Two options exist for reconstruction after brachytherapy. With the traditional reconstruction, or "immediate reconstruction technique," a flap is placed over the wound, which is then closed. In some cases, direct closure of the wound is performed. The second option is staged reconstruction, which involves use of a temporary closure (e.g., wound vacuum) that facilitates the start of radiotherapy as soon as a day after surgery without creating problems related to wound healing (Naghavi et al., 2016).

Use of CT simulation permits radiation planning, where the goal is to treat the tumor bed at risk and spare exposure of healthy bone, muscle, nerves, and other tissues. The radioactive seed(s) is inserted through the catheters and are positioned at different points in the tumor bed, resulting in a conformal radiation dose. In HDR brachytherapy, the seed is delivered by means of an afterloader device, said Dr. Naghavi.

Advances in technology have greatly reduced the toxicity associated with radiation therapy. Conventional treatment involved a large radiation field that left many patients with lifelong painful lymphedema. Improvement in surgical and radiation oncology techniques have facilitated the use of smaller treatment fields. The radiated area decreased from a 10-cm margin around the tumor bed before 1990 to 4 cm today, said Dr. Naghavi.

Impaired wound healing is the most common toxicity associated with radiation therapy, occurring in 15% to 40% of patients. Other notable adverse effects include edema (~20%) and fibrosis and associated decreases in range of motion (~20%). Less common toxicities include bone fracture (2%–10%), peripheral nerve injury (1%–10%), and secondary malignancy (< 1%/year). The overall incidence of radiation therapy–associated toxicity is about 1% per year.

Several clinical practices and precautions can mitigate the risk of radiation-associated toxicity, said Dr. Naghavi. Risk mitigation begins with appropriate patient selection. For example, patients with peripheral vascular disease or diabetes mellitus have an increased risk of wound complications with preoperative radiation therapy.

Use of wound vacuums and flaps, as well as avoiding flap exposure during radiation treatment (O’Sullivan et al., 2013) has been shown to help reduce the risk of acute radiation-induced toxicity. Reducing the time from preoperative radiation therapy to surgery also mitigates the risk of complications. An interval of 6 to 8 weeks or less is associated with fewer complications (Griffin et al., 2015).

With regard to mitigation of long-term toxicities, minimizing the radiation field can help. Larger field size is associated with an increased risk of fibrosis, joint stiffness, and edema. Several studies have shown that improved targeting of radiation therapy can reduce long-term sequelae, including use of image guidance (Wang et al., 2015), conformal treatment (Folkert et al., 2014), and concise treatment volumes (Wang et al., 2015). Reduced-dose radiotherapy helps minimize pain, edema, decreased range of motion (Stinson et al., 1991), and the risk of fracture.

## OPTIONS FOR SYSTEMIC THERAPY

Until the 1970s, the STS therapeutic armamentarium comprised surgery and radiation therapy. That changed in 1973 with the publication of a landmark study demonstrating activity with doxorubicin in a variety of malignancies, including STS (O’Bryan et al., 1973).

Until about 2000, the emergence of doxorubicin remained the major development in systemic therapy for STS. During that time, considerable clinical research focused on combination therapy with various conventional chemotherapy agents, said Ms. Clark. The only other notable development came in the form of ifosfamide in 1997, which proved active in STS but demonstrated greater activity in osteosarcomas.

A series of serendipitous developments toward the end of the 1990s constituted a turning point in systemic therapy for STS. Perhaps the key development was the emergence of the tyrosine kinase inhibitor imatinib (Gleevec) as a breakthrough treatment for chronic myeloid leukemia. Coincident with that development, unrelated studies showed that gastrointestinal stromal tumors (GISTs), long thought to be related to leiomyosarcomas, appeared to originate from interstitial cells of Cajal. More important, many GISTs were found to express C-KIT and CD34, potential targets of imatinib.

"This is the key that kind of unlocked our thinking for sarcomas, and we began to think, ’What is happening genetically with these tumors? Where can we block the cell differentiation to stop tumor growth?’ " said Ms. Clark.

The accumulation of evidence eventually led to a clinical trial evaluating two different doses of imatinib in patients with unresectable or metastatic GISTs (Demetri et al., 2002). More than half the patients attained partial responses, and 41% had stable disease. The treatment also led to a meaningful improvement in survival as compared with historical data, said Ms. Clark.

The benefits of imatinib come with a substantial amount of toxicity. Most patients develop edema, as many as half have skin rash, and a majority have gastrointestinal effects, including nausea, diarrhea, vomiting, and anorexia. About a third of patients treated with imatinib have elevated liver enzymes, and almost half have increased serum creatinine at some point.

The next developments in systemic therapy for STS would not come for about another decade. The multi–tyrosine kinase inhibitor pazopanib (Votrient) received FDA approval in 2012 for patients with STS previously treated with chemotherapy.

Supporting evidence for the approval came primarily from the phase III PALETTE trial involving 370 patients with advanced, nonadipocytic GIST (van der Graaf et al., 2012). Patients were randomized 2:1 to pazopanib or placebo, and the trial had a primary end point of progression-free survival (PFS). The results showed that patients assigned to pazopanib had a median PFS of 4.6 months compared with 1.6 months for the placebo group (*p* < .0001).

About half of patients treated with pazopanib develop fatigue, diarrhea, nausea, and weight loss associated with loss of appetite. Between a third and 40% of patients develop hypertension, anorexia, hair hypopigmentation, and vomiting. Prominent hematologic toxicity includes leukopenia in more than 40% of patients and thrombocytopenia in a third or more.

In 2015, trabectedin (Yondelis), a new-generation alkylating agent, gained FDA approval for unresectable or metastatic liposarcoma or leiomyosarcoma previously treated with an anthracycline. In a randomized phase III trial (Demetri et al., 2016), the agent failed to improve overall survival (the primary endpoint) vs. dacarbazine but led to significantly better median PFS (4.1 vs. 1.5 months, *p* < .001).

The principal toxicity associated with trabectedin is nausea, which can be severe. Use of antiemetic regimens from the first dose can make the nausea more manageable.

"If you don’t work very hard in controlling it from the first dose, patients get a learned response, and then it’s very difficult for subsequent doses," said Ms. Clark.

In 2016, eribulin (Halaven) gained FDA approval for the treatment of metastatic liposarcoma previously treated with at least two prior chemotherapy regimens, including an anthracycline. Eribulin has the distinction of being the first systemic therapy to demonstrate improved overall survival vs. an active control in a phase III trial.

Single-agent eribulin was compared with dacarbazine in patients with previously treated liposarcoma or leiomyosarcoma (Schöffski et al., 2016). The trial involved 452 patients, and the results showed a 2-month improvement in median overall survival for patients randomized to eribulin (13.5 vs. 11.5 months, *p* = .0169). Subgroup analysis showed the overall results were driven by the patients with liposarcoma, who had a median overall survival of 15.6 months with eribulin vs. 8.4 months with dacarbazine. Prominent toxicities associated with eribulin include nausea in 35% to 40% of patients, constipation in a third, and neutropenia and anemia in 60% to 80% of patients.

In October 2016, the FDA granted accelerated approval for olaratumab (Lartruvo; Tap et al., 2015), a monoclonal antibody directed against PDGF receptor-α. The agent inhibits PDGF ligand binding and cellular signaling associated with cell proliferation, angiogenesis, and fibroblast recruitment. The FDA granted olaratumab breakthrough therapy status for STS in combination with doxorubicin.

In a phase Ib/II randomized trial, the addition of olaratumab to doxorubicin failed to improve the primary endpoint of PFS compared with doxorubicin alone, but showed a trend toward improvement (6.6 vs. 4.1 months, *p* = .0615). The combination resulted in significant improvement in the secondary endpoint of overall survival (26.5 vs. 14.7 months, *p* = .0003). The principal toxicities associated with olaratumab were fatigue, alopecia, hyperglycemia, neutropenia, thrombocytopenia, and musculoskeletal pain, all occurring in a majority of patients treated with the drug.

"So, where do we go from here?" Ms. Clark said. "When you think about the timeline, you see these big breaks. We see 10 years, 12 years, 20 years in between. Let’s just hope that since we started in 2012 we are on a roll. There are now clinical trials for sarcoma that involve vaccines. We are looking at immunotherapy. We are doing intratumoral immunotherapy. We are looking at CAR T-cell therapy, and we are looking at tyrosine kinase inhibitors in combination with other targeted therapy and investigational agents. Let’s hope that the long desert is behind us."
